# Supplementary material for: Depressive symptoms and their association with age, chronic conditions and health status among middle-aged and elderly people in peri-urban Tanzania
Source: Glob Ment Health (Camb). 2023 May 4;10:e27. doi: 10.1017/gmh.2023.17 (PMC10579685; doi:10.1017/gmh.2023.17)
Supplement: Supplementary file 1 [file S2054425123000171sup001.docx]

**Supplementary material**

**Table S1:** Age structure of the HAALSI Tanzania study sample and the Dar Es Salaam Urban Cohort Study

(a) Men

|  | **DUCS 2019–20** | **DUCS 2016** | **HAALSI TZ (selected)** | **HAALSI TZ (analyzed)** |
| --- | --- | --- | --- | --- |
| **Age group** | | | | |
| 40–44 years | 5090 (31.4) | 3004 (33.1) | 716 (29.2) | 132 (18.7) |
| 45–49 years | 3715 (22.9) | 1972 (21.7) | 559 (22.8) | 143 (20.3) |
| 50–54 years | 2513 (15.5) | 1348 (14.9) | 382 (15.6) | 97 (13.8) |
| 55–59 years | 1628 (10) | 1069 (11.8) | 305 (12.4) | 99 (14) |
| 60–64 years | 1246 (7.7) | 717 (7.9) | 190 (7.8) | 83 (11.8) |
| 65–69 years | 860 (5.3) | 467 (5.2) | 154 (6.3) | 70 (9.9) |
| 70–74 years | 521 (3.2) | 242 (2.7) | 72 (2.9) | 38 (5.4) |
| 75+ years | 646 (4) | 248 (2.7) | 72 (2.9) | 43 (6.1) |
| Total | 16219 (100) | 9067 (100) | 2450 (100) | 705 (100) |
|  | | | | |
| **P** | **Comparison** | | | |
| <0.001 |  |  | X | X |
| <0.001 |  | X |  | X |
| <0.001 | X |  |  | X |
| 0.018 |  | X | X |  |
| 0.001 | X |  | X |  |
| <0.001 | X | X |  |  |

(b) Women

|  | **DUCS 2019–20** | **DUCS 2016** | **HAALSI TZ (selected)** | **HAALSI TZ (analyzed)** |
| --- | --- | --- | --- | --- |
| **Age group** | | | | |
| 40–44 years | 4965 (34.4) | 2808 (35.9) | 796 (33.2) | 467 (30.8) |
| 45–49 years | 3289 (22.8) | 1545 (19.7) | 525 (21.9) | 337 (22.2) |
| 50–54 years | 1892 (13.1) | 1222 (15.6) | 360 (15) | 236 (15.6) |
| 55–59 years | 1431 (9.9) | 789 (10.1) | 251 (10.5) | 171 (11.3) |
| 60–64 years | 925 (6.4) | 570 (7.3) | 175 (7.3) | 131 (8.6) |
| 65–69 years | 644 (4.5) | 335 (4.3) | 110 (4.6) | 78 (5.1) |
| 70–74 years | 426 (2.9) | 210 (2.7) | 64 (2.7) | 31 (2) |
| 75+ years | 880 (6.1) | 352 (4.5) | 119 (5) | 64 (4.2) |
| Total | 14452 (100) | 7831 (100) | 2400 (100) | 1515 (100) |
|  | | | | |
| **P** | **Comparison** | | | |
| 0.353 |  |  | X | X |
| 0.002 |  | X |  | X |
| <0.001 | X |  |  | X |
| 0.194 |  | X | X |  |
| 0.031 | X |  | X |  |
| <0.001 | X | X |  |  |

(..) = % of total. DUCS = Dar Es Salaam Urban Cohort Study, HAALSI = Health and Aging in Africa: Longitudinal Studies in three INDEPTH Communities, TZ = Tanzania. In similar pairwise comparisons of samples and populations, the sex structure differed between HAALSI (analyzed), HAALSI (selected), DUCS 2016 and DUCS 2019–20 (all P ≤ 0.002) but not between DUCS 2016 and DUCS 2019–20 (P = 0.104).

**Table S2:** Assessment of chronic conditions and health status

| **Chronic condition** | **Assessment** | **Definition** |
| --- | --- | --- |
| Anemia | 1. Hemoglobin point-of-care test  2. Self-report of smoking | Hemoglobin < 12 mg/dl; threshold adjustments for smoking (+0.3 mg/dl) and African origin (−1 mg/dl) |
| Signs of cognitive problems | Self-rated present memory, immediate and delayed word recall of 10 nouns, date, and president naming; scoring of correct answers; adapted from US Health and Retirement Study | Present memory rated as fair or poor, score ≤ 1.5 standard deviation of population mean |
| Chronic cough | Self-reported coughing | Self-report of usually coughing and not having tuberculosis as a chronic condition |
| Signs of depression | 10-item Centre for Epidemiological Studies Depression Scale (CES-D-10) | CES-D-10 score ≥ 10 |
| Diabetes | 1. Blood glucose point-of-care test  2. Self-report of current treatment | Blood glucose ≥ 200 mg/dl, fasting blood glucose ≥ 126 mg/dl, or currently on treatment for diabetes with diet, weight loss, pills, or insulin injection |
| Kidney disease | Self-reported diagnosis | Ever diagnosed with kidney disease or low kidney function |
| HIV | Self-reported diagnosis | Ever tested HIV positive |
| Hyper-cholesterolemia | Self-reported diagnosis | Ever diagnosed with high cholesterol |
| Hypertension | 1. Blood pressure was measured according to World Health Organization standards up to three times, with a few minutes in between each time; the mean of all measurements was calculated  2. Self-report of current treatment | Systolic pressure ≥ 140 mmHg, diastolic pressure ≥ 90 mmHg or currently on treatment for hypertension |
| Ischemic heart disease | 1. Self-reported diagnosis of angina, heart failure, or heart attack  2. Modified Rose Angina Questionnaire | Rose Angina Questionnaire criteria, ever received diagnosis of angina, or ever received diagnosis of heart attack |
| Obesity | Measured weight and height | Body mass index > 30 kg/m^2^ |
| Signs of sarcopenia | Measured grip strength | Grip strength < 27 kg for man, grip strengths < 16 kg for women |
| Stroke | 1. Self-reported diagnosis  2. Self-reported treatment | Ever diagnosed with stroke, mini-stroke, transient ischemic attack; suddenly lost half vision, or ever received treatment for stroke |
| Tuberculosis | 1. Self-reported diagnosis  2. Self-reported treatment | Ever diagnosed with tuberculosis, but never received treatment, or currently on tuberculosis treatment |
| Underweight | Measured weight and height | Body mass index < 18.5 kg/m^2^ |

Self-reported diagnoses were obtained by asking: Have you ever been told by a doctor, nurse, or other healthcare worker that you have had … ? Modified Rose Angina Questionnaire criteria included whether experienced any pain or discomfort in the chest or pain going to the left arm or neck when walking uphill or hurrying during the last 12 months; stopping, slowing down, or carrying on after taking a pain-relieving medicine that dissolves in the mouth (e.g., a nitro spray or tablet) when getting pain or discomfort while walking; and pain or discomfort is relieved when standing still.

**Table S3:** Sociodemographic characteristics and substance use of study participants

|  | **Total** | **Male** | **Female** | **P** |
| --- | --- | --- | --- | --- |
|  | **N ≤ 2220** | **N ≤ 705** | **N ≤ 1515** |  |
| **Socioeconomic factors** | | | | |
| Age (years), n = 2220 | 50 (44–59) | 53 (46–63) | 49 (43–57) | <0.001 |
| Country of origin, n = 2220 |  |  |  | 0.45 |
| Tanzania | 2197 (99.0) | 696 (98.7) | 1501 (99.1) |  |
| Other | 23 (1.0) | 9 (1.3) | 14 (0.9) |  |
| Religion, n = 2220 |  |  |  | 0.67 |
| Islam | 1202 (54.1) | 377 (53.5) | 825 (54.5) |  |
| Christianity | 1018 (45.9) | 328 (46.5) | 690 (45.5) |  |
| Marital status, n = 2220 |  |  |  | <0.001 |
| Married or cohabitant | 1575 (70.9) | 619 (87.8) | 956 (63.1) |  |
| Widowed | 381 (17.2) | 40 (5.7) | 341 (22.5) |  |
| Never married or separated | 264 (11.9) | 46 (6.5) | 218 (14.4) |  |
| Number of children, n = 2220 |  |  |  | 0.002 |
| 0 | 63 (2.8) | 14 (2.0) | 49 (3.2) |  |
| 1–2 | 499 (22.5) | 132 (18.7) | 367 (24.2) |  |
| 3+ | 1658 (74.7) | 559 (79.3) | 1099 (72.5) |  |
| Can read and/or write, n = 2220 | 1864 (84.0) | 648 (91.9) | 1216 (80.3) | <0.001 |
| Formal education, n = 2220 |  |  |  | <0.001 |
| 0–6 school years | 1725 (77.7) | 480 (68.1) | 1245 (82.2) |  |
| 7–10 school years | 108 (4.9) | 52 (7.4) | 56 (3.7) |  |
| 10+ school years | 387 (17.4) | 173 (24.5) | 214 (14.1) |  |
| Work status, n = 2220 |  |  |  | <0.001 |
| Homemaker | 759 (34.2) | 85 (12.1) | 674 (44.5) |  |
| Working | 1041 (46.9) | 416 (59.0) | 625 (41.3) |  |
| Not working | 420 (18.9) | 204 (28.9) | 216 (14.3) |  |
| No food in house, n = 2220 |  |  |  | <0.001 |
| Never | 1143 (51.5) | 412 (58.4) | 731 (48.3) |  |
| Rarely (once or twice) | 626 (28.2) | 185 (26.2) | 441 (29.1) |  |
| Sometimes (3–10 times) | 171 (7.7) | 41 (5.8) | 130 (8.6) |  |
| Often (more than 10 times) | 280 (12.6) | 67 (9.5) | 213 (14.1) |  |
| Household wealth index (0–100), n = 2177 | 18 (15–21) | 18 (16–22) | 18 (15–21) | <0.001 |
| Ward, N = 2,220 |  |  |  | 0.76 |
| Ukonga (with 4 major streets) | 1224 (55.1) | 392 (55.6) | 832 (54.9) |  |
| Gongolamboto (with 3 major streets) | 996 (44.9) | 313 (44.4) | 683 (45.1) |  |
| **Substance use** | | | | |
| Currently smoking, n = 2218 | 96 (4.3) | 84 (11.9) | 12 (0.8) | <0.001 |
| Ever smoked, n = 2218 | 296 (13.3) | 247 (35.1) | 49 (3.2) | <0.001 |
| Signs of alcohol problems, n = 2218 | 172 (7.8) | 92 (13.1) | 80 (5.3) | <0.001 |

n (%) or median (IQR).

**Table S4:** Depressive symptoms, chronic conditions, and healths status of study participants

|  | **Total** | **Male** | **Female** | **P** |
| --- | --- | --- | --- | --- |
|  | **N ≤ 2220** | **N ≤ 705** | **N ≤ 1515** |  |
| **Depressive symptoms** | | | | |
| CES-D-10 score, n = 2220 | 7 (5–11) | 6 (4–11) | 7 (5–11) | 0.006 |
| CES-D-10 score ≥ 10, n = 2220 | 700 (31.5) | 209 (29.6) | 491 (32.4) | 0.19 |
| **Chronic condition** | | | | |
| Hypertension, n = 2168 | 1108 (51.1) | 373 (54.4) | 735 (49.6) | 0.038 |
| Anemia, n = 977 | 333 (34.1) | 84 (28.7) | 249 (36.4) | 0.019 |
| Obesity, n = 2126 | 688 (32.4) | 109 (16.1) | 579 (39.9) | <0.001 |
| Diabetes, n = 978 | 307 (31.4) | 98 (33.4) | 209 (30.5) | 0.36 |
| Ischemic heart disease, n = 2219 | 264 (11.9) | 45 (6.4) | 219 (14.5) | <0.001 |
| Signs of cognitive problems, n = 2213 | 145 (6.6) | 47 (6.7) | 98 (6.5) | 0.86 |
| HIV, n = 2209 | 112 (5.1) | 13 (1.9) | 99 (6.6) | <0.001 |
| High cholesterol, n = 2216 | 110 (5.0) | 24 (3.4) | 86 (5.7) | 0.023 |
| Stroke, n = 2218 | 107 (4.8) | 31 (4.4) | 76 (5.0) | 0.53 |
| Tuberculosis, n = 2216 | 107 (4.8) | 29 (4.1) | 78 (5.2) | 0.29 |
| Underweight, n = 2126 | 89 (4.2) | 37 (5.5) | 52 (3.6) | 0.042 |
| Chronic cough, n = 2215 | 73 (3.3) | 18 (2.6) | 55 (3.6) | 0.18 |
| Kidney disease, n = 2216 | 64 (2.9) | 15 (2.1) | 49 (3.2) | 0.15 |
| **Multimorbidity** | | | | |
| No chronic condition | 103 (10.9) | 41 (14.6) | 62 (9.4) |  |
| 2+ chronic conditions, n = 941 | 575 (61.1) | 150 (53.4) | 425 (64.4) | 0.002 |
| **Health status** | | | | |
| Health today, n = 2218 |  |  |  | <0.001 |
| Good or very good | 840 (37.9) | 307 (43.5) | 533 (35.2) |  |
| Moderate | 1161 (52.3) | 333 (47.2) | 828 (54.7) |  |
| Bad or very bad | 217 (9.8) | 65 (9.2) | 152 (10.0) |  |
| Limitations in activities of daily living, n = 2219 | 373 (16.8) | 90 (12.8) | 283 (18.7) | <0.001 |

n (%) or median (IQR).

**Table S5:** Estimated prevalence of depressive symptoms among 40+ year-old people in the Ukonga and Gongolamboto wards in the Dar es Salaam region

| **Prevalence of depressive symptoms** | **All** | **Men** | **Women** | **P** |
| --- | --- | --- | --- | --- |
| **Age group** | | | | |
| 40+ years, n = 2220 | 30.7 (28.5–32.9) | 28.8 (25.3–32.4) | 32.9 (30.4–35.3) | 0.067 |
| 40–44 years, n = 599 | 25.5 (21.3–29.6) | 22.7 (15.6–29.9) | 28.3 (24.2–32.4) | 0.189 |
| 45–49 years, n = 480 | 33.6 (28.9–38.4) | 34.3 (26.5–42.1) | 32.9 (27.9–38.0) | 0.779 |
| 50–54 years, n = 333 | 31.4 (25.6–37.3) | 29.9 (20.7–39.1) | 33.5 (27.4–39.5) | 0.522 |
| 55–59 years, n = 270 | 27.9 (22.3–33.6) | 24.2 (15.7–32.8) | 32.2 (25.1–39.2) | 0.160 |
| 60–64 years, n = 214 | 26.8 (20.5–33.2) | 24.1 (14.8–33.4) | 30.5 (22.6–38.5) | 0.301 |
| 65–69 years, n = 148 | 33.3 (25.5–41.2) | 34.3 (23.0–45.6) | 32.1 (21.5–42.6) | 0.775 |
| 70+ years, n = 176 | 48.0 (40.4–55.7) | 43.5 (32.0–54.9) | 52.1 (41.8–62.4) | 0.269 |
| **Chronic condition** | | | | |
| Ischemic heart disease, n = 264 | 59.6 (53.1–66.1) | 64.2 (49.2–79.2) | 57.6 (50.9–64.3) | 0.432 |
| Tuberculosis, n = 107 | 48.6 (37.3–60.0) | 41.5 (21.7–61.3) | 55.3 (43.9–66.7) | 0.235 |
| Signs of cognitive problems, n = 145 | 46.2 (37.3–55.2) | 46.1 (30.9–61.4) | 46.3 (35.9–56.8) | 0.982 |
| Stroke, n = 107 | 44.8 (34.4–55.2) | 57.6 (39.3–75.8) | 35.4 (24.3–46.5) | 0.042 |
| Chronic cough, n = 73 | 40.6 (27.9–53.3) | 47.2 (22.5–71.9) | 36.3 (22.8–49.8) | 0.442 |
| HIV, n = 112 | 38.6 (27.8–49.4) | 39.7 (11.5–68.0) | 38.1 (28.2–48.0) | 0.913 |
| Obesity, n = 688 | 33.6 (29.7–37.5) | 35.2 (25.8–44.6) | 32.9 (29.0–36.8) | 0.659 |
| Anemia, n = 333 | 33.4 (27.8–39.0) | 29.6 (19.3–39.9) | 36.1 (30.0–42.2) | 0.283 |
| Diabetes, n = 307 | 32.4 (26.4–38.5) | 37.7 (27.4–48.0) | 27.1 (20.9–33.2) | 0.083 |
| Kidney disease, n = 64 | 32.3 (19.2–45.3) | 26.4 (3.06–49.7) | 36.5 (22.0–51.0) | 0.462 |
| Hypertension, n = 1108 | 31.4 (28.3–34.5) | 29.9 (25.0–34.9) | 33.1 (29.5–36.6) | 0.317 |
| High cholesterol, n = 110 | 31.1 (20.5–41.6) | 43.7 (22.0–65.4) | 23.3 (14.1–32.5) | 0.089 |
| Underweight, n = 89 | 28.6 (18.1–39.2) | 22.1 (7.98–36.2) | 40.1 (26.0–54.2) | 0.077 |
| **Morbidity** | | | | |
| No chronic condition, n = 103 | 23.0 (12.8–33.3) | 24.9 (9.87–39.9) | 19.7 (9.75–29.7) | 0.570 |
| 2+ chronic conditions, n = 575 | 34.1 (29.6–38.5) | 33.7 (25.6–41.8) | 34.4 (29.8–39.0) | 0.880 |
| **Health status** | | | | |
| Health today |  |  |  |  |
| Bad or very bad, n = 217 | 43.0 (35.5–50.5) | 45.8 (32.5–59.0) | 40.7 (32.5–48.9) | 0.524 |
| Moderate, n = 1161 | 35.0 (31.9–38.2) | 34.9 (29.4–40.4) | 35.1 (31.8–38.5) | 0.943 |
| Good or very good, n = 840 | 23.3 (20.1–26.6) | 20.8 (16.0–25.5) | 27.3 (23.4–31.2) | 0.037 |
| Limitations in activities of daily living, n = 373 | 40.2 (34.5–45.8) | 45.2 (34.1–56.4) | 37.0 (31.1–42.9) | 0.202 |

% (95% CI). Estimates weighted to match age and sex structure in the Dar Es Salaam Urban Cohort Study 2019–20.

**Table S6:** Association between depressive symptoms and age, chronic conditions, and health status among 40+ year-old people in the Ukonga and Gongolamboto wards in the Dar es Salaam region

| **Depressive symptoms (CES-D-10 ≥ 10), n = 2220** | **Univariable regressions** | **Multivariable regression** | **Multivariable regression with control variables** |
| --- | --- | --- | --- |
| **Age** | | | |
| Age group (40–44 years) | 1 | 1 | 1 |
| 45–49 years | 1.35 (1.04–1.75)^*^ | 1.37 (1.04–1.8)^*^ | 1.35 (1.01–1.81)^*^ |
| 50–54 years | 1.29 (0.97–1.73) | 1.23 (0.9–1.68) | 1.13 (0.80–1.59) |
| 55–59 years | 1.12 (0.81–1.53) | 1.04 (0.74–1.45) | 0.94 (0.65–1.36) |
| 60–64 years | 1.05 (0.74–1.49) | 0.97 (0.67–1.41) | 0.83 (0.55–1.26) |
| 65–69 years | 1.34 (0.91–1.97) | 1.18 (0.76–1.83) | 0.88 (0.53–1.46) |
| 70+ years | 2.35 (1.66–3.33)^***^ | 1.88 (1.26–2.8)^**^ | 1.29 (0.81–2.06) |
| **Chronic condition** | | | |
| Ischemic heart disease | 3.43 (2.64–4.46)^***^ | 3.01 (2.26–4.01)^***^ | 2.73 (2.01–3.71)^***^ |
| Tuberculosis | 2.42 (1.64–3.57)^***^ | 2.12 (1.36–3.3)^***^ | 2.02 (1.24–3.27)^**^ |
| Signs of cognitive problems | 1.90 (1.35–2.67)^***^ | 1.37 (0.93–2.02) | 1.30 (0.86–1.95) |
| Stroke | 1.56 (1.05–2.32)^*^ | 1.16 (0.76–1.79) | 1.15 (0.71–1.86) |
| Chronic cough | 1.29 (0.79–2.09) | 1.05 (0.63–1.76) | 1.13 (0.63–2.01) |
| HIV | 1.31 (0.88–1.94) | 1.18 (0.77–1.8) | 0.94 (0.57–1.55) |
| Obesity | 1.16 (0.96–1.41) | 1.25 (0.97–1.6) | 1.28 (0.97–1.69) |
| Anemia | 1.32 (1.01–1.71)^*^ | 1.18 (0.86–1.62) | 1.13 (0.80–1.61) |
| Diabetes | 1.07 (0.82–1.40) | 1.02 (0.75–1.4) | 0.99 (0.70–1.39) |
| Kidney disease | 1.15 (0.68–1.94) | 0.95 (0.54–1.68) | 1.25 (0.69–2.26) |
| Hypertension | 1.00 (0.83–1.19) | 0.91 (0.72–1.15) | 0.81 (0.63–1.04) |
| High cholesterol | 0.78 (0.50–1.20) | 0.57 (0.34–0.94)^*^ | 0.56 (0.34–0.94)^*^ |
| Underweight | 1.19 (0.77–1.85) | 1.05 (0.67–1.67) | 0.93 (0.55–1.57) |
| Multimorbidity | 1.53 (1.23–1.90)^***^ | 0.99 (0.68–1.43) | 1.04 (0.70–1.55) |
| **Health status** | | | |
| Health today (bad or very bad) | 1 | 1 | 1 |
| Moderate | 0.79 (0.58–1.06) | 0.95 (0.68–1.31) | 0.95 (0.67–1.34) |
| Good or very good | 0.48 (0.35–0.66)^***^ | 0.66 (0.46–0.94)^*^ | 0.71 (0.48–1.05) |
| Limitations in activities of daily living | 1.35 (1.07–1.70)^*^ | 1.04 (0.8–1.36) | 0.90 (0.67–1.21) |
| **Substance use** | | | |
| Currently smoking | 1.04 (0.67–1.61) |  | 1.04 (0.58–1.87) |
| Ever smoked | 1.19 (0.92–1.54) |  | 1.04 (0.72–1.51) |
| Signs of alcohol problems | 0.99 (0.71–1.39) |  | 0.91 (0.61–1.37) |
| **Socioeconomic factors** | | | |
| Sex (male) | 1 |  | 1 |
| Female | 1.14 (0.94–1.38) |  | 0.94 (0.71–1.25) |
| Religion (Islam) | 1 |  | 1 |
| Christianity | 0.93 (0.78–1.12) |  | 1.03 (0.83–1.28) |
| Country of origin (Tanzania) | 1 |  | 1 |
| Other | 0.95 (0.39–2.32) |  | 1.14 (0.39–3.35) |
| Marital status (married or cohabitant) | 1 |  | 1 |
| Widowed | 1.66 (1.32–2.09)^***^ |  | 1.43 (1.06–1.93)^*^ |
| Never married or separated | 1.24 (0.94–1.64) |  | 1.07 (0.77–1.48) |
| Number of children (none) | 1 |  | 1 |
| 1–2 children | 1.27 (0.72–2.25) |  | 1.10 (0.57–2.10) |
| 3+ children | 1.01 (0.58–1.75) |  | 0.88 (0.47–1.67) |
| Literacy | 0.62 (0.49–0.78)^***^ |  | 0.90 (0.66–1.22) |
| Formal education (0–6 school years) | 1 |  | 1 |
| 7–10 school years | 1.31 (0.88–1.96) |  | 1.35 (0.83–2.18) |
| 10+ school years | 0.85 (0.67–1.08) |  | 0.88 (0.65–1.20) |
| Work status (homemaker) | 1 |  | 1 |
| Other work | 0.86 (0.70–1.06) |  | 1.38 (1.07–1.79)^*^ |
| Not working | 1.72 (1.34–2.20)^***^ |  | 2.09 (1.52–2.87)^***^ |
| No food in house in the past year (never) | 1 |  | 1 |
| Rarely | 0.97 (0.78–1.22) |  | 0.97 (0.75–1.25) |
| Sometimes | 2.60 (1.87–3.61)^***^ |  | 2.44 (1.68–3.53)^***^ |
| Often | 4.27 (3.25–5.61)^***^ |  | 3.45 (2.51–4.76)^***^ |
| Household wealth index (0–100) | 0.95 (0.87–1.04) |  | 1.04 (0.92–1.16) |
| Ward/street (Ukonga/Markaz) | 1 |  | 1 |
| Ukonga/Mazizini | 0.56 (0.37–0.85)^**^ |  | 0.52 (0.33–0.82)^**^ |
| Ukonga/Mongolandege | 0.84 (0.54–1.32) |  | 0.84 (0.51–1.37) |
| Ukonga/Mwembe Madafu | 0.30 (0.20–0.45)^***^ |  | 0.28 (0.18–0.43)^***^ |
| Gongolamboto/Gongolamboto | 0.84 (0.55–1.29) |  | 0.59 (0.37–0.93)^*^ |
| Gongolamboto/Guluka Kwalala | 0.53 (0.34–0.84)^**^ |  | 0.48 (0.29–0.80)^**^ |
| Gongolamboto/Ulongoni | 0.18 (0.12–0.28)^***^ |  | 0.20 (0.13–0.32)^***^ |
| Constant | Yes | 0.35 (0.23–0.53)^***^ | 0.64 (0.26–1.57) |

OR (95% CI). Estimates weighted to match age and sex structure in the Dar Es Salaam Urban Cohort Study 2019–20.

**Figure S1:** Age structure of the HAALSI Tanzania study sample and the Dar Es Salaam Urban Cohort Study

| (a) Men | (b) Women |
| --- | --- |
|  |  |

DUCS = Dar Es Salaam Urban Cohort Study, HAALSI = Health and Aging in Africa: Longitudinal Studies in three INDEPTH Communities. The analyzed HAALSI Tanzania study sample included 705 men and 1515 women aged 40+ years. In the DUCS 2016, 9067 (21.1%) of 42,914 men and 7831 (17.1%) of 45,843 were aged 40+ years. In the DUCS 2019–20, 16,219 (26.8%) of 60,485 of the men and 14,452 (21.3%) of 67,832 were aged 40+ years in 2019–20. The study sample was weighted to match the age and sex structure of the DUCS 2019–20.
